# Bioengineered synthesis of phytochemical-adorned green silver oxide (Ag_2_O) nanoparticles via *Mentha pulegium* and *Ficus carica* extracts with high antioxidant, antibacterial, and antifungal activities

**DOI:** 10.1038/s41598-022-26021-4

**Published:** 2022-12-13

**Authors:** Maryam Shahzad Shirazi, Mahdi Moridi Farimani, Alireza Foroumadi, Kamal Ghanemi, Maurizio Benaglia, Pooyan Makvandi

**Affiliations:** 1 Department of Phytochemistry, Medicinal Plants and Drugs Research Institute, Shahid Beheshti University, Tehran, Iran; 2 Department of Medicinal Chemistry, Faculty of Pharmacy, Tehran University of Medical Sciences, Tehran, Iran; 3 Department of Marine Chemistry, Faculty of Marine Science, Khorramshahr University of Marine Science and Technology, Khorramshahr, Iran; 4 Dipartimento di Chimica, Università degli Studi di Milano, Via Golgi, 19, 20133 Milan, Italy; 5 Centre for Materials Interfaces, Istituto Italiano di Tecnologia, viale Rinaldo Piaggio 34, 56025 Pontedera, Pisa Italy

**Keywords:** Biocatalysis, Quantum dots

## Abstract

Silver oxide nanoparticles have various biomedical and pharmaceutical applications. However, conventional nanofabrication of Ag_2_O is associated with the use of toxic chemicals and organic solvents. To circumvent this hurdle, herein silver oxide quantum dots (Ag_2_O-QDs) were synthesized quickly (3 min) via the use of ultrasonic irradiation and plant-extract. Additionally, due to ultrasonic irradiation's effect on cell-wall destruction and augmentation of extraction efficiency, ultrasonic was also used in the preparation of *Mentha pulegium* and *Ficus carica* extracts (10 min, r.t) as natural eco-friendly reducing/capping agents. The UV–Vis result indicated a broad absorption peak at 400–500 nm. TEM/SEM analysis showed that ultrasound introduced a uniform spherical particle and significantly reduced particle size compared to the conventional heating method (∼ 9 nm vs. ∼ 100 nm). Silver and oxygen elements were found in the bio-synthesized Ag_2_O by EDS. The FTIR and phenol/flavonoid tests revealed the presence of phenol and flavonoid associated with the nanoparticles. Moreover, nanoparticles exhibited antioxidant/antibacterial/antifungal activities. The MIC and MBC results showed the Ag_2_O QDs synthesized with *M. pulegium* extract have the highest antibacterial activity against *E. coli* (MBC = MIC:15.6 ppm), which were significantly different from uncoated nanoparticles (MBC = MIC:500 ppm). The data reflects the role of phyto-synthesized Ag_2_O-QDs using ultrasonic-irradiation to develop versatile and green biomedical products.

## Introduction

In contrast to other transition metal nanoparticles, Ag_2_O nanoparticles have received a great deal of attention because of their wide applications in bioimaging, formulation of ointments, lotions, mineral-based sunscreens, drug delivery^1^, antioxidant, antifungal^2^, antibacterial^3^, anticancer, antiviral, anti-inflammatory, biosensor, human health care^4^, cytotoxic^5^, and wound healing^6^.

Since physical and chemical methods to synthesize and stabilize Ag_2_O nanoparticles are harmful and expensive, green methods are more attractive because they are simple, cheap, and non-toxic. These methods use biological resources such as plants, fungi, and bacteria. The use of these renewable resources, including plants (inactivated plant tissues, plant extracts and living plants) in phytosynthesis of nanoparticles reduces the need to use toxic chemicals and solvents and makes them ideal for use in biomedical applications^7–9^. Plant extracts are non-toxic, cheap and easily accessible. They have a wide range of metabolites that contribute to the bio-reduction of metal ions and act as effective phyto-reducing, stabilizing and capping agents for a variety of metals. Therefore, they are valuable alternatives for the large-scale production of metal nanoparticles^10–12^. Plant-produced NPs are more stable and distinct in shape and size than conventional chemical methods, along with showing antioxidant activity^12^.

*Mentha pulegium* is an aromatic plant in the *Lamiaceae* family, widely distributed in nature and commonly known as pennyroyal^13,14^. It has strong antibacterial properties against many gram-negative and positive bacteria^13^. It also has antigenotoxic, antioxidative, anti-inflammatory, antiseptic, and analgesic properties, and is used as a condiment and as a medicine to treat gastrointestinal disorders^14,15^.

*Ficus carica* belongs to the plant family *Moraceae,* which is widespread in tropical and subtropical countries^16^. *F. carica* fruits, which are cholesterol-free edible fruits, have polyphenols and polysaccharides and are used as medicine. These polysaccharides have antitumor, antispasmodic, antioxidant, and anti-inflammatory properties^17^. This plant is rich in dietary fiber, protein, organic acids, minerals, different vitamins, carbohydrates, phenolic compounds, and anthocyanins, which are considered to be potent molecules to treat various diseases^17,18^.

The ultrasonic irradiation source creates unique reaction conditions that do not exist in other methods^19,20^. Compared to other methods, ultrasonic irradiation-based methods are more favorable for milder conditions, more uniform size distribution, higher surface area, faster reaction rates, higher efficiency, shorter reaction times, and improved phase purity^20^. In addition to using green ultrasound irradiation for synthesis, it can also be used for extraction, which reduces solvent consumption and improves plant cell wall destruction as well as the mass transfer of analytes (such as phenolic compounds or antioxidants) into an aqueous solution^21^.

Herein, ultrasonic irradiation as a safe, green, and simple method was used to extract *M. pulegium* leaves as well as *F. carica* fruits and synthesize Ag_2_O quantum dots. Ultrasound-assisted synthesis (UAS) of Ag_2_O QDs was carried out in a plant-mediated approach using the aqueous extracts of *M. pulegium* and *F. carica* obtained through the ultrasound-assisted extraction (UAE) technique. Furthermore, different techniques were used to evaluate the antibacterial, antifungal, and antioxidant activities of the extracts and Ag_2_O quantum dots.

## Materials and methods

### Plant materials

*Mentha pulegium* leaves and *F. carica fruits* of superior quality were purchased from a herbal medicine shop in Ahvaz, Iran, and identified by the plant taxonomist Dr. A. Sonboli. Voucher specimens (No. MPH-2721 and MPH-2722, respectively) were deposited in the Herbarium of the Medicinal Plants and Drugs Research Institute (MPDRI), Shahid Beheshti University, Tehran, Iran. The collection of plant material and related studies complies with relevant institutional, national, and international guidelines and legislation.

### Chemicals and bacterial strains

All chemicals and materials needed for synthesis were purchased from Merck and Sigma companies. Also, materials needed for biological experiments, as well as the bacterial strains including *Pseudomonas aeruginosa* (ATCC 27853), *Staphylococcus aureus* (ATCC 25923), *Bacillus subtilis* (ATCC 465), *Escherichia coli* (ATCC 1399), *Candida albicans* (ATCC 10231), and *Aspergillus oryzae* (ATCC 1101), were procured from the Pasture Institute of Iran, Iranian biological resource center, and Iranian research organization for science & technology (IROST).

### Ultrasound-assisted extraction (UAE)

The fruits of *F. carica* and *M. pulegium* leaves were washed using distilled water to remove dust particles and any other impurities. They were then dried at 50 °C for 12 h. Then, they were powdered by a mechanical grinder and stored.

To prepare *M. pulegium* leaves extract (MPE) and *F. carica* fruit extract (FCE), 10 g of previously prepared powders was added to 80 mL of deionized water. The mixture was then placed in the ultrasonic device (40 kHz) at room temperature for 10 min, and finally, the crude extract was filtered through whatman paper.

### Chemical and green ultrasound-assisted synthesis (UAS) of Ag_2_O QDs

The UAS/phyto-synthesized Ag_2_O quantum dots (Ag_2_O QDs) were prepared by bio-reducing AgNO_3_ under ultrasonic irradiation (WUC-D, Korea) in the presence of aqueous *M. pulegium* and *F. carica* extracts^22^. To do this, 12.5 mL of aqueous extract was mixed with 25 mL of 26 × 10^–4^ mol L^−1^ AgNO_3_ solution, and the mixture was immersed in the ultrasonic bath (40 kHz, r.t) until no further color change was observed in the solution. It was confirmed that the phyto-reduction of Ag^+^ ions to Ag^0^ takes place completely within 3 min by varying the initial color of the reaction mixture. Then, the suspension was centrifuged (10 min, 6000 rpm) and washed with double distilled water (DDW). A vacuum dryer was used to dry the precipitated Ag_2_O QDs (45 °C, 24 h). As a result, Ag_2_O QDs were synthesized from *F. carica* extract (UAS/FCE-Ag_2_O QDs) as well as *M. pulegium* extract (UAS/MPE-Ag_2_O QDs) using the UAS method.

According to the literature^23^, the Ag_2_O nanoparticles were also chemically synthesized (Chem-syn/Ag_2_O NPs). First, the aqueous solution of 5 × 10^–3^ mol L^−1^ AgNO_3_ (80 mL) was prepared and stirred with a magnetic stirrer at 60 °C. Adding 20 mL of NaOH_(aq)_ solution (25 × 10^–3^ mol L^−1^) immediately produced a gray-yellow suspension containing a large amount of silver hydroxide (AgOH) precipitate. Since AgOH particles are thermodynamically unstable, they are chemically turned into Ag_2_O particles^23^.

### Characterization of NPs

The optical behavior of samples was measured using UV–Vis absorption spectra (Shimadzu, UV-2501PC, Japan), and their functional groups were identified by Fourier-transform infrared spectra (Bruker Tensor 27, 500–4000 cm^–1^, KBr wafer). The SEM sputter coater (Coxem SPT-20, South Korea) was utilized for coating conductive materials (Au/Pd) onto the surface of the SEM samples. The size, morphology, and elemental analysis of the quantum dots were then identified by scanning electron microscopy/energy-dispersive X-ray spectroscopy (SEM–EDS, FEI ESEM Quanta 200, EDS Silicon Drift 2017, USA). In addition, identification of phase purity and structural information of the sample was obtained using X-ray diffraction (XRD) (PHILIPS, PW1730, Netherlands) diffractometer with Cu Kα source (λ = 1.54056 Å). Microplate reader BioTek (Epoch 2) was used to read biological data.

### Total phenolic and flavonoid content

The total phenol content in the samples was assessed using the Folin-Ciocalteu colorimetric assay^24^, with some modifications. In this method, Folin–Ciocalteu’s phenol reagent (125 µL) and Na_2_CO_3_ solution (100 µL, 7.5% w/v) were first added to each sample (25 µL). The mixture was then incubated (25 °C, 2 h, and dark). The absorbance of all samples was measured at 760 nm. Gallic acid was used as a standard, and the results were expressed as gallic acid equivalents (µg GAE/mg sample).

The samples' total flavonoid content was quantified using an aluminium chloride colorimetric method as previously reported^24^. Firstly, aliquots of 25 μL of each sample, DDW (100 μL), and sodium nitrite (7.5 μL, 5% w/v NaNO_2_) solution were mixed. The resulting mixture was incubated for 6 min. Then, aluminium chloride (7.5 μL, 10% w/v AlCl_3_), 100 μL of sodium hydroxide (4% w/v NaOH) solution, and DDW (10 μL) were added to the mixture and kept in the dark (r.t, 15 min). Finally, the absorbance of the mixture was recorded at 510 nm using a UV–Vis spectrophotometer. Quercetin was used as a standard, and the results were expressed as quercetin equivalents (µg QU/mg sample)^25^. The total phenol and flavonoid content was quantified five times for each sample.

### Antioxidant activities

#### DPPH assay

According to the literature^24^, the scavenging activity of the samples against the DPPH (2,2-Diphenyl-1-picrylhydrazyl) radical was determined with some modifications. 50 μL of each sample was mixed with 200 μL of DPPH methanolic solution (0.0788 mg/mL) and set aside in the dark (30 min, r.t). Ascorbic acid was used as a positive control. The ability to scavenge DPPH radical was measured at 517 nm, as follows:

1 $${\text{Radical scavenging}}\left( \% \right) = \left[ {\left( {{\text{A}}_{0} - {\text{ A}}_{{1}} } \right)/{\text{A}}_{0} } \right] \times 100$$

Where A_0_ and A_1_ are the absorbance intensities of the control and the sample, respectively. DPPH assay was repeated five times for each sample.

#### ABTS assay

The ABTS radical scavenging activity of the samples was evaluated using the previously reported method^26^. Firstly, ABTS_(aq)_ (7 mM) solution and potassium persulfate solution (2.45 mM) were mixed and then left to dark (r.t, 24 h), to generate radical cation (ABTS^⋅+^). The ABTS radical cation is stable at room temperature in the dark for more than 2 days. The solution was diluted with PBS to obtain a final absorbance of 0.700 ± 0.02 at 734 nm and equilibrated at 30 °C. Then, 290 μL of ABTS solution was treated with 10 μL of each sample. The chemical changes were monitored by a colorimetric method at 734 nm. The ABTS radical-scavenging activity was calculated according to Eq. (1). ABTS assay was repeated 5 times for each sample.

#### Ferric ion reducing antioxidant power assay

The ferric ion reducing antioxidant power (FRAP) assay was performed according to the literature^27^, with slight modifications, using a standard solution of FeSO_4_⋅7H_2_O (0.0625–1 mM). The stock FRAP reagent contained FeCl_3_⋅6H_2_O (20 mM, 2.5 mL), TPTZ solution (10 mM, 2.5 mL), and acetate buffer (300 mM, 25 mL, pH = 3.6) in 40 mM HCl. The fresh FRAP reagent (2.5 mL) was added to the sample (1 mL) at 37 °C for 10 min. The absorption was recorded at 593 nm. The final results were expressed as mM ferrous/mg of sample. The FRAP assay was repeated five times for each sample.

### Antibacterial activities

The antimicrobial and antifungal activities of plant extracts and quantum dots synthesized by biological and chemical methods were evaluated using the Disc Diffusion Method (DDM), the minimum inhibitory concentration (MIC), and the minimum bactericidal concentration (MBC) against gram-positive bacteria such as *Staphylococcus aureus* and *Bacillus subtilis,* as well as gram-negative bacteria like *Escherichia coli* and *Pseudomonas aeruginosa*.

The Disc Diffusion Method (DDM), was used to evaluate the in vitro antibacterial potential of aqueous extracts and Ag_2_O QDs against bacteria strains, according to the literature^28^. In summary, 0.1 mL of each organism was first dispersed using a sterile swab on the Muller Hinton Agar medium. After preparing different concentrations (12.5, 25, and 50 mg/mL) of the samples, the filter paper discs (diameter: 6 mm) were loaded with the different concentrations of samples and placed on the plates. After incubation (37 °C, 24 h), the diameter of the inhibition zone (mm) surrounding the disc was used to evaluate the samples' antimicrobial activity.

The minimum inhibitory concentration (MIC) value is the lowest concentration of the sample that inhibits the visible growth of bacteria^29^. This assay was performed according to the previous study^30^. In this method, different concentrations of extracts as well as Ag_2_O QDs samples (0.488 to 1000 ppm) were mixed and inoculated with Mueller–Hinton broth which was exposed to different test organism suspensions. After an incubation period (37 °C, 24 h), the concentration of the last well that shows no macroscopic growth defined the MIC value.

According to the literature^30^, the minimum bactericidal concentration (MBC) was directly determined from MIC wells and by sub-culturing diluted sample solutions (with no turbidity or growth) onto Muller Hinton Agar. After incubation (37 °C, 24 h), the minimum concentration of the sample which can kill the bacteria on this solid medium was reported as the MBC values.

### Antifungal activity

The antifungal activity of the extracts and Ag_2_O quantum dots was evaluated using a disc diffusion technique, as previously reported^31^, with slight modifications. To perform this test, two different fungi, including *Aspergillus oryzae* and *Candida albicans,* were selected. Then, the filter paper discs were placed on the plates after loading with the different concentrations of samples (12.50, 25, and 50 mg/mL) and were then incubated (29 °C, 72 h). The inhibition zone (mm) was measured and recorded as the antifungal activity of the samples.

## Result and discussion

### Characterization of Ag_2_O QDs

**Figure 1:**
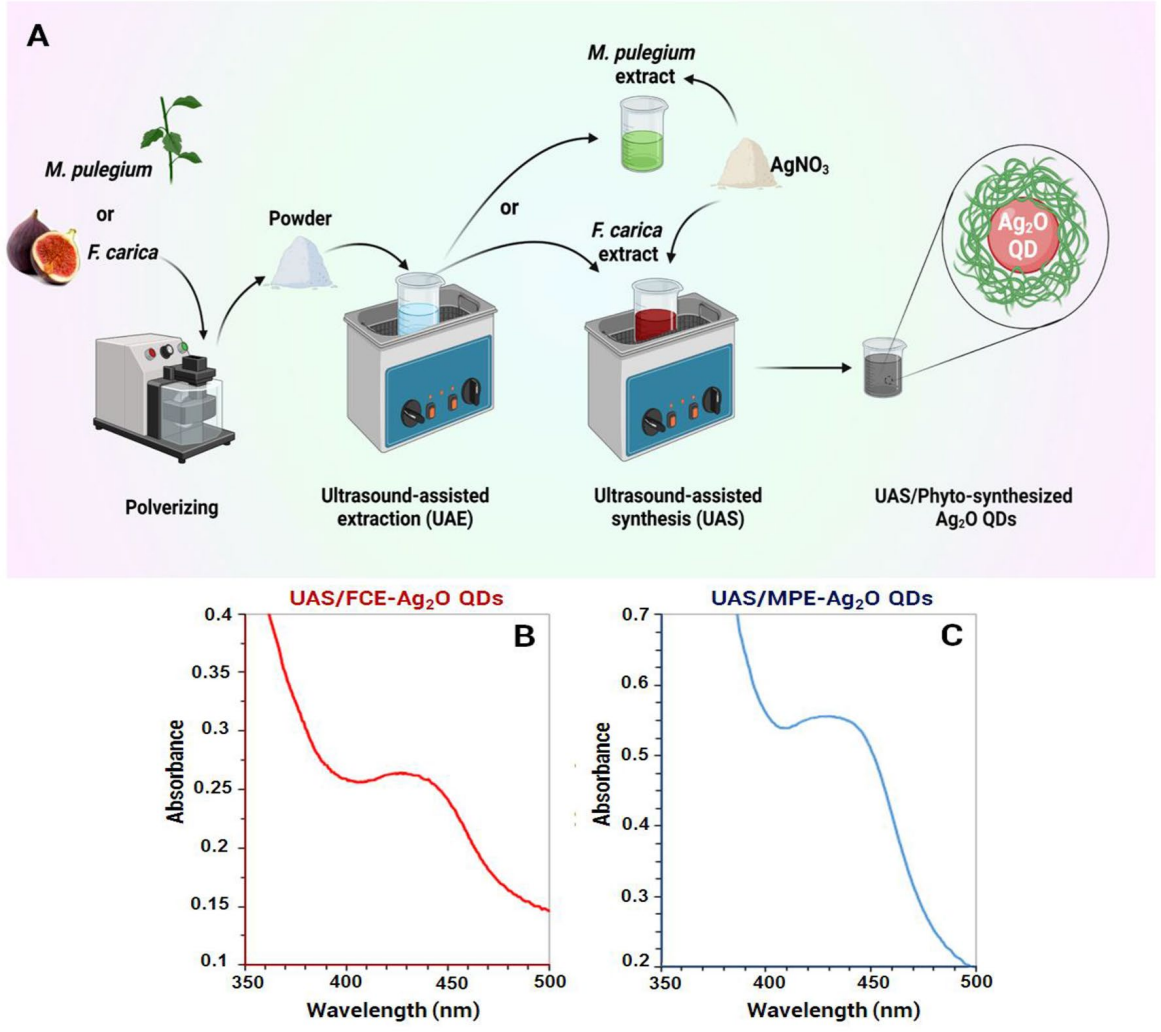
Schematic illustration of synthesis approach of Ag_2_O-QDs (**A**); the UV–Vis spectra of the UAS/phyto-synthesized Ag_2_O-QDs (**B**,**C**).

In this study, Ag_2_O QDs were synthesized with ultrasonic irradiation, plant extract, and silver nitrate solution as a metal salt precursor (Fig. 1A)**.** The *M. pulegium* and *F. carica* extracts were employed as reducing, capping, and stabilizing agents. Ultrasound irradiation has several advantages in the extraction process, including reducing solvent consumption, increasing plant cell wall destruction, and mass transferring active components (e.g., phenolic compounds) into solution^21^. In addition, the sonosynthesis method led to the very rapid formation of monodisperse and fine Ag_2_O QDs.

The reduction process was initially assessed visually by changing the color and turbidity of the solutions containing Ag^+^ ions and FCE or MPE, after ultrasound irradiation. It was confirmed that the phyto-reduction of Ag^+^ ions to Ag^0^ takes place completely by varying the color of the reaction mixture.

A UV–Vis analysis was used to investigate the optical behavior and preliminary characterization of QDs. Figure 1B,C exhibits the spectra of the green synthesized Ag_2_O QDs obtained from the corresponding extracts under ultrasound irradiation within the range of 3**5**0–5**0**0 nm. The Ag_2_O QDs biosynthesized by MPE and FCE led to a broad absorption band between 400 and 500 nm. This confirms the existence of Ag_2_O QDs in the mixture and is in line with earlier reports^32–35^.

**Figure 2:**
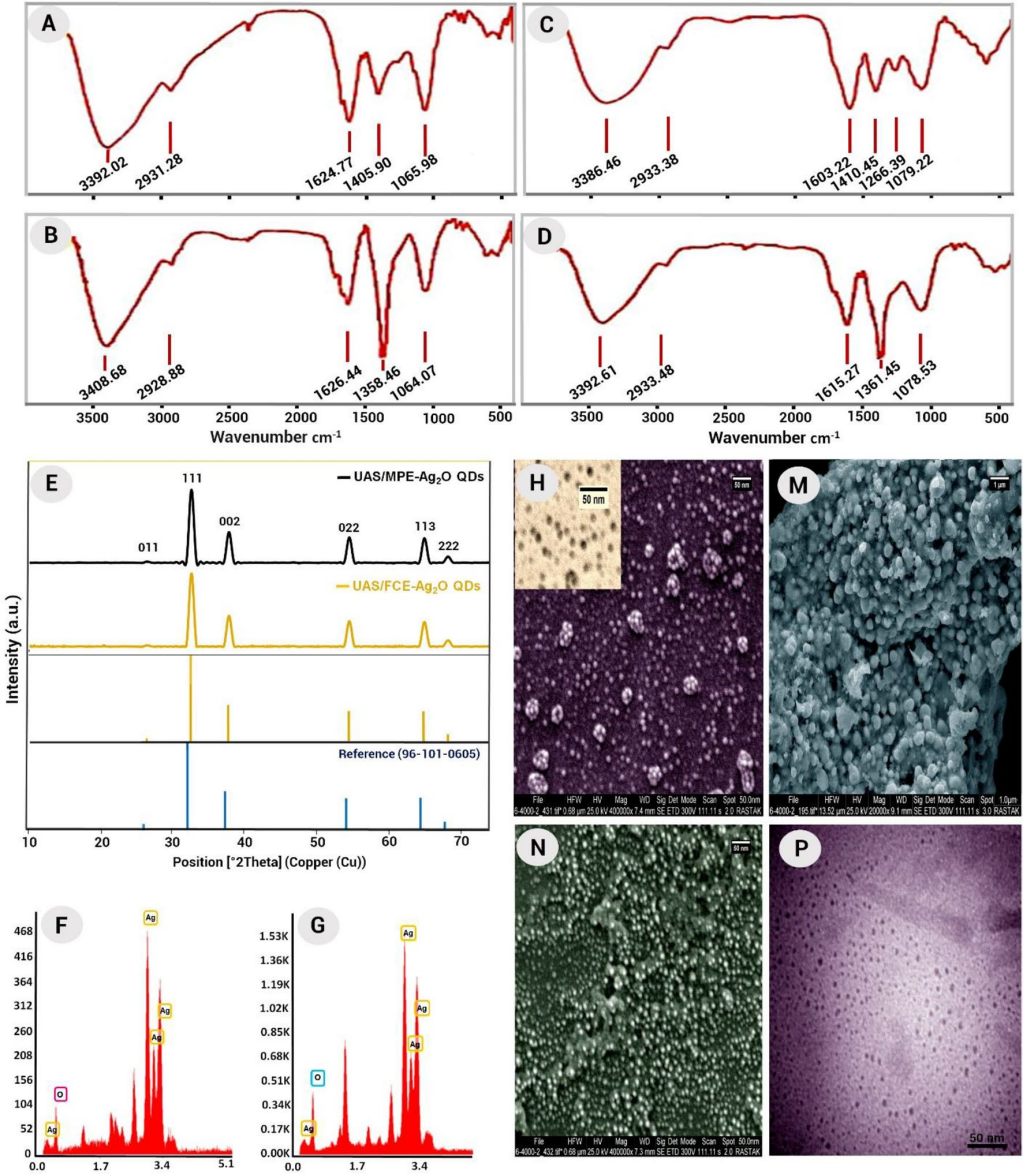
The FTIR spectra of FCE (**A**), UAS/FCE-Ag_2_O QDs (**B**), MPE (**C**), and UAS/MPE-Ag_2_O QDs (**D**); XRD patterns (**E**), EDX spectra and SEM images of UAS/FCE-Ag_2_O QDs (**G**,**H**) and UAS/MPE-Ag_2_O QDs using ultrasound (**F**,**N**) respectively; SEM image of *M. pulegium-mediated* synthesis of Ag_2_O QDs with the conventional heating method without ultrasound (M) and TEM image of UAS/MPE-Ag_2_O nanoparticles (**P**).

Since the stability of quantum dots is very critical, due to the adhesion of metabolites in plant extracts to the surface of quantum dots, they were used for in situ bio-capping and the stability of Ag_2_O NPs^34,36^. Given that the stabilizing or capping agents attached to the Ag_2_O QDs’ surface change the FTIR spectrum, it provides useful information about the surface chemistry of QDs (Fig. 2A–D)^37,38^. The control spectrum (FCE) reflects the complex nature of biological materials (Fig. 2A). In this spectrum, the strong peak at 3392 cm^−1^ is due to the stretching vibration of the OH groups. Furthermore, the absorption peaks at 2931, 1624, 1406, and 1065 cm^−1^ can be attributed to the stretching vibration of the C–H methyl/methylene bond, C=O of ester or carboxylic acid, C–O bond of ester/ether, and the C–N/C–O of aliphatic amines or alcohol/phenol, respectively^39,40^. The presence of these functional groups on nanoparticles' surfaces increases their stability and biological efficiency^41^. As shown in Fig. 2B, after the reaction of the FCE with silver ions and the formation of particulates, there was a shift in the peaks. These shifts indicate the binding of extract phytochemicals as coating and stabilizing agents to the surface of the quantum dots^38,39^. It is known that biological components reduce metal salts through their functional groups and form nanoparticles^39,40^.

The same results were observed for MPE and Ag_2_O QDs phyto-synthesized from it (Fig. 2C,D). All these peaks show the presence of phytochemicals including flavonoids, polyphenolics, amino acids, etc., which have different functional groups as bio-reducing, bio-capping, and bio-stabilizing agents in the synthesis of UAS/phyto-synthesized Ag_2_O QDs and prevent aggregation of quantum dots^42^.

To study the crystal structures and phase purity of the UAS/plant-synthesized Ag_2_O QDs, X-ray diffraction spectroscopy (XRD) was deployed. The XRD patterns (Fig. 2E) of sonochemically prepared UAS/MPE-Ag_2_O QDs and UAS/FCE-Ag_2_O QDs indicated a wide range of 10° ≤ 2θ ≤ 80° which confirms the successful bio-synthesis of Ag_2_O-QDs with a high crystalline single phase. As found, the XRD spectra of samples had similar diffraction peaks. Several strong Bragg reflections in XRD patterns were presented at 2θ = 26.64, 32.55, 37.76, 54.48, 64.92, and 68.19 which were related to the (011), (111), (002), (022), (113), and (222) planes, respectively. These known and intense peaks confirm the cubic phase structure (reference code: 96-101-0605; Fig. 2E) of both phyto-synthesized Ag_2_O QDs using UAS, as reported in previous researches^43–45^. In addition, the crystal size of the Ag_2_O QDs was calculated from the XRD pattern employing the Scherrer equation. The particles' size of ~ 9 nm was determined.

The SEM–EDS micrographs show an overview of the size, morphology, and elemental/chemical bulk quantification of the samples. SEM images of UAS/FCE-Ag_2_O QDs and UAS/MPE-Ag_2_O QDs synthesized using ultrasound irradiation are shown in Fig. 2H,N as well as MPE-Ag_2_O nanoparticles prepared using the conventional heating method (without ultrasound) in Fig. 2M. Figure 2H,N illustrates that ultrasonic irradiation produces spherical Ag_2_O QDs with a narrow size distribution and regular shape. But conventional phyto-synthesis of Ag_2_O nanoparticles using *M. pulegium* extract (MPE) produced larger spherical (~ 100 nm) and aggregated nanoparticles (Fig. 2M). TEM image (Fig. 2P) analysis also revealed the spherical nanoparticles formed by MPE*.* To our knowledge, there are no very fine phyto-synthesized Ag_2_O-QDs with a very narrow size distribution in the literature.

The presence of Ag and O elements in both phyto-synthesized Ag_2_O QDs was confirmed from their signals in the EDX spectrum, Fig. 2F,G. Other signals are due to the presence of phytochemicals in the extracts that capped the surface of the quantum dots^42^.

### Total phenol and flavonoid contents

**Figure 3:**
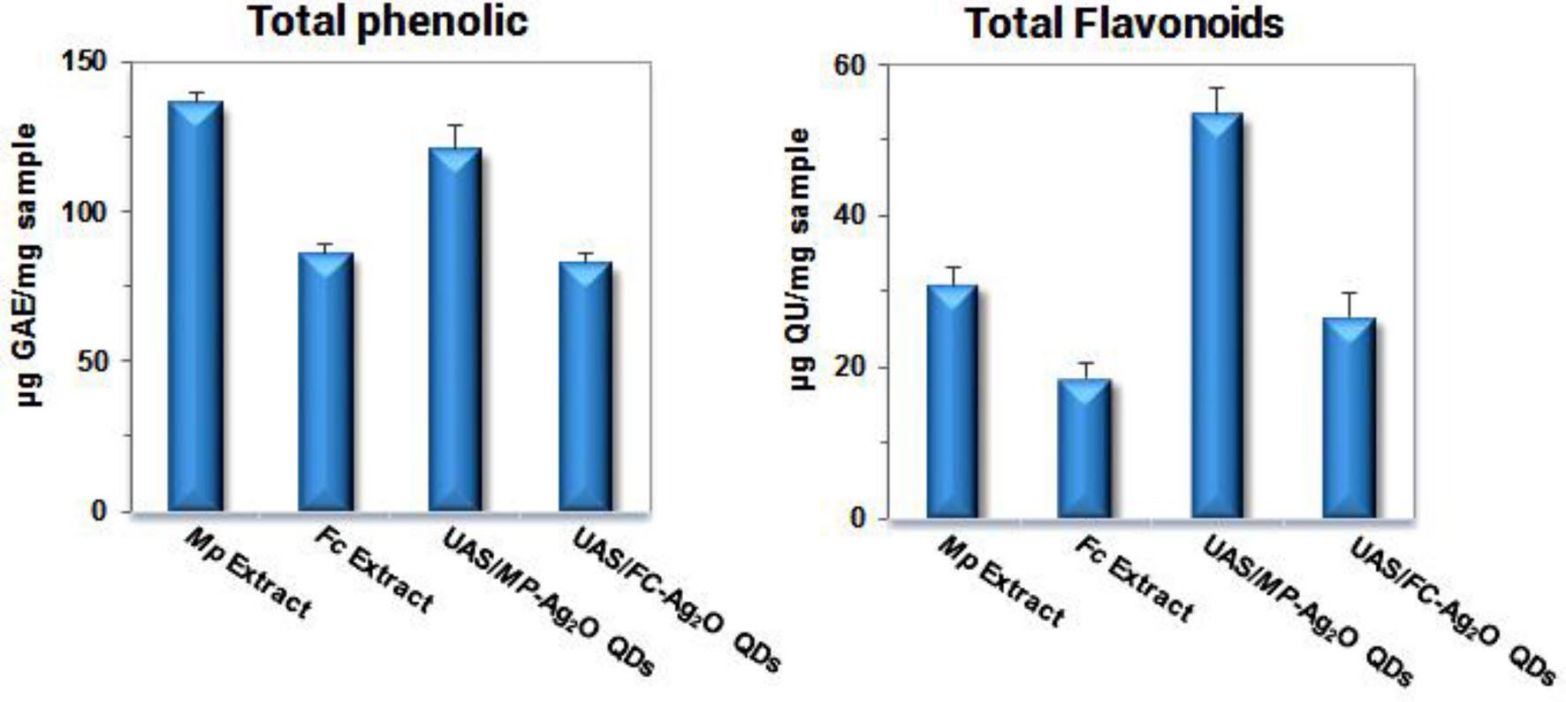
Total phenol and flavonoid contents of Ag_2_O QDs and extracts.

**Figure 4:**
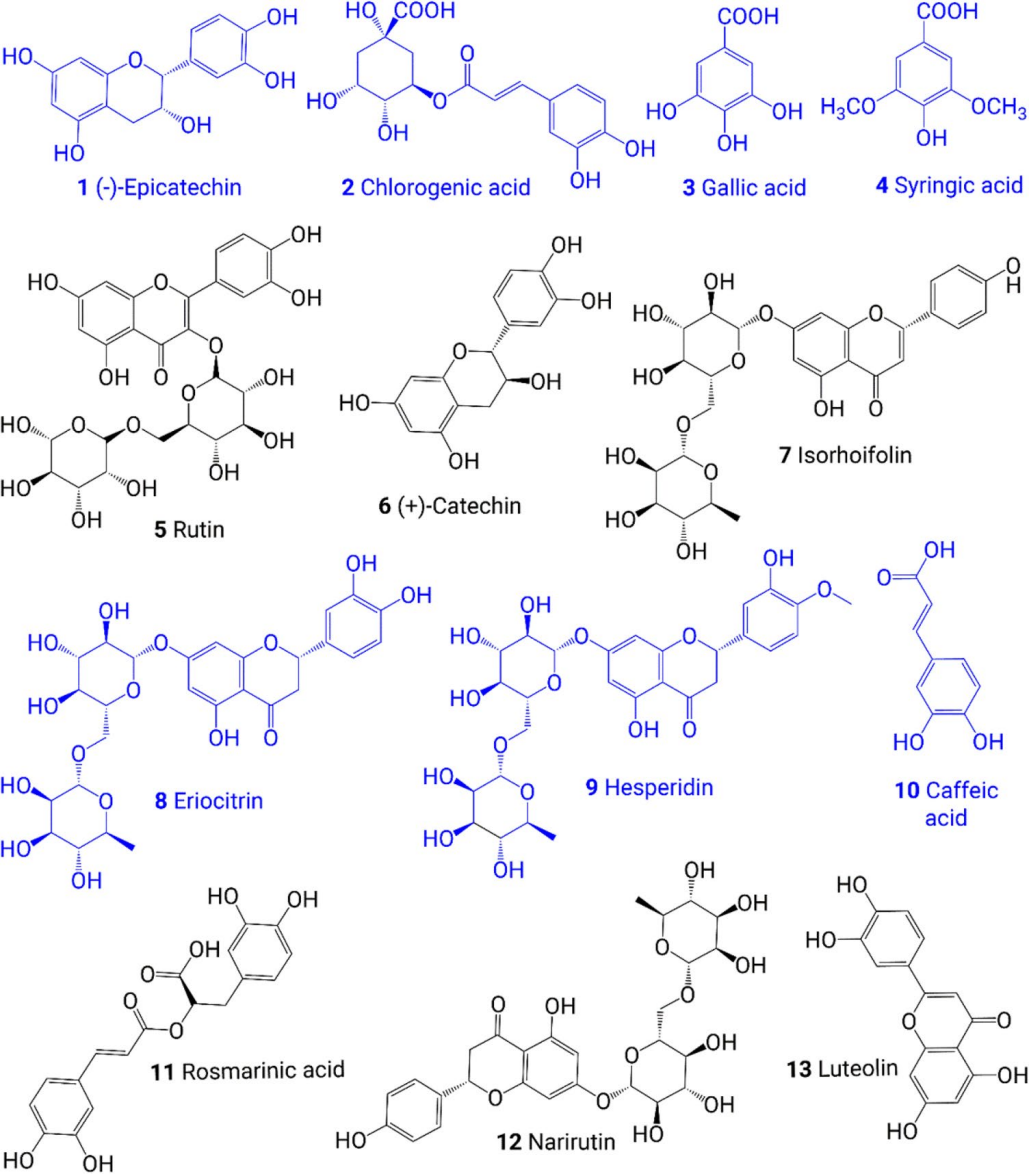
Bioactive compounds in *F. carica* (**1**–**6**) and *M. pulegium* (**7**–**13**).

The total phenolic and flavonoid contents in the crude extracts and phyto-synthesized QDs were analyzed (Fig. 3). Some phenols and flavonoids in *F. carica*^46^ and *M. pulegium*^47^ plants are shown in Fig. 4. The biological effects of plant extracts depend on their components, such as phenolic and flavonoid contents, solvent type, polarity, and extraction method^48^. The phenolic compounds are a class of bioactive components in plants that have a wide range of biological properties, including antifungal, antibacterial, anti-inflammatory, antiviral, antiallergic, and antiviral properties^48^.

As shown in Fig. 3, the amount of phenolic compounds in all samples is significant, and the total phenolic contents of samples are in the following order: MPE > UAS/MPE-Ag_2_O QDs, as well as FCE > UAS/FCE-Ag_2_O QDs. As can be seen, extracts showed higher phenol content than extracts containing quantum dots. This can be attributed to the phenolic compounds´ involvement in the reduction of silver ions as well as their absence in the reduction process of molybdenum and tungsten ions in Fulin reagent. In contrast to total phenols, quantum dots-bearing extracts have a higher level of total flavonoids (Fig. 3), possibly as a result of silver ions participating in chelate formation with flavonoids. Therefore, the chelating potential of flavonoids is confirmed by measuring the flavonoid content of quantum dots^49^.

### Antioxidant activity

**Figure 5:**
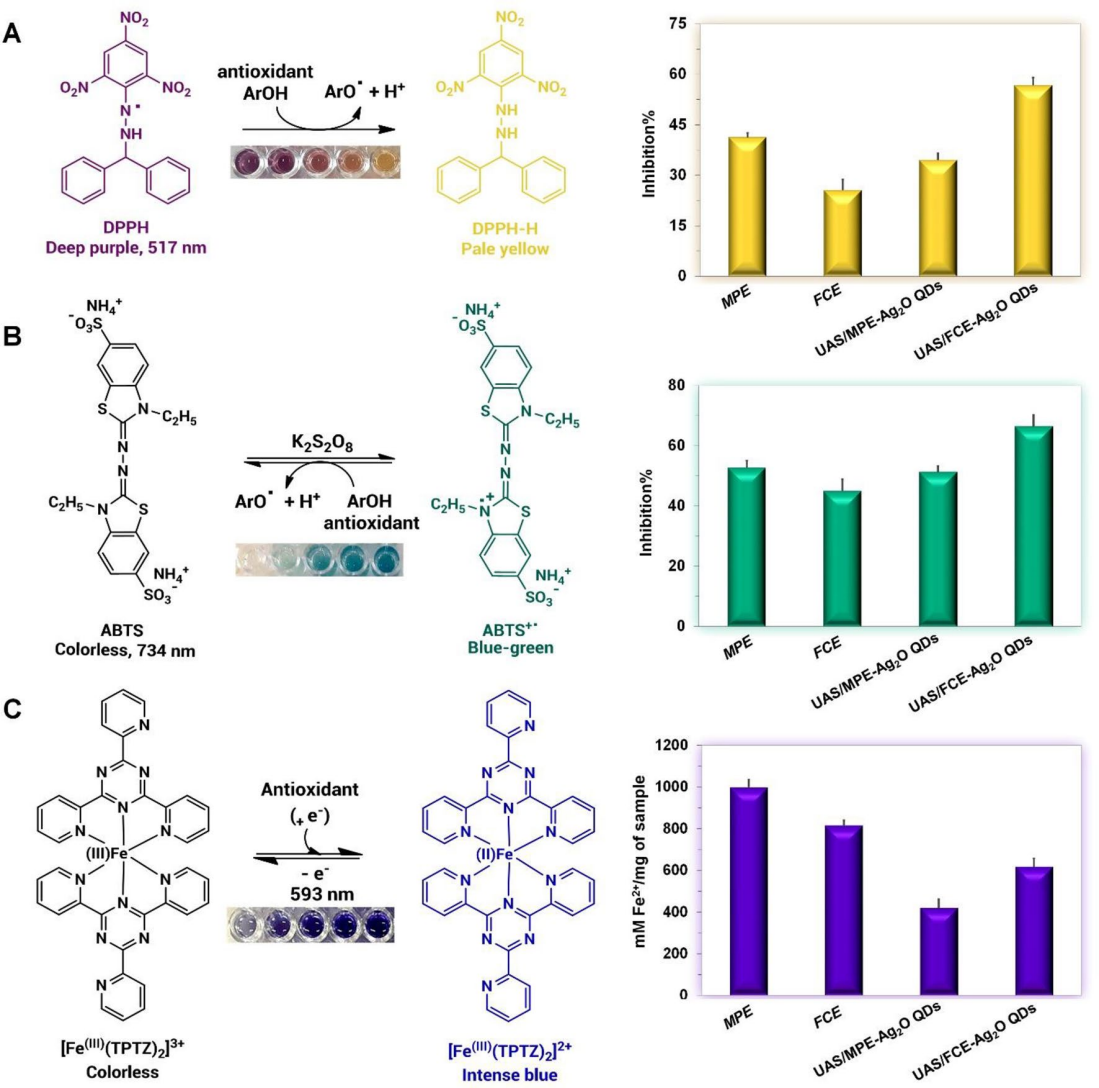
DPPH (**A**), ABTS (**B**), FRAP (**C**) reaction mechanisms with an antioxidant agent. Different colors indicate different concentrations of the desired sample.

Free radicals produced in the body cause hundreds of diseases, but they are usually controlled by the body's antioxidant defense system. This is because antioxidants neutralize or end the chain reaction started by free radicals^33^. Phenolic compounds have antioxidant activity due to their ability as reducing agents, radical scavengers, and hydrogen donors. Since they can donate hydrogen atoms from their aromatic hydroxyl groups to free radicals and have a resonance effect in their aromatic rings, they are excellent antioxidants^50^. In general, antioxidant activity is often attributed to the total phenol content and sometimes to the synergistic or antagonistic effects of compounds in crude extracts as well as the chemical structures of the compounds^51^. Additionally, phytochemical compounds on the surface of the synthesized QDs can improve antioxidant activity^52^. During this study, different assays, including DPPH, ABTS, and ferric-reducing power (FRAP) methods, were employed to determine the antioxidant ability of the plant extracts and their corresponding QDs. The reaction mechanism between these oxidants and antioxidants is shown in Fig. 5^53^. The antioxidant properties of the samples to neutralize the stable free chromogenic radical (2,2-diphenyl-1-picrylhydrazyl) DPPH^⋅^^54^ and the cation radical ABTS^⋅+^ (2,2′-Azino-bis(3-ethylbenzothiazoline-6-sulfonic acid)^26^ were evaluated in the DPPH and ABTS assays, respectively. The cation radicals of ABTS^⋅+^ are more reactive than DPPH radicals and are used to estimate both the lipophilic and hydrophilic antioxidant activity^55^.

According to the DPPH and ABTS results (Fig. 5A,B), the maximum inhibition percentage values occurred in the UAS/FCE-Ag_2_O QDs (56.72% for DPPH and 66.43% for ABTS). Notably, the UAS/FCE-Ag_2_O QDs have better antioxidant activity than FCE. These results are in line with the reports of Maheshwaran et al.^45^ and Abdel-Aziz et al.^56^. The obtained antioxidant activity confirmed the inhibition potential of biosynthesized Ag_2_O QDs against reactive oxygen species (ROS)^45^. As seen, the antioxidant activity of UAS/FCE-Ag_2_O QDs does not follow the phenol content trend. Thus, the synergistic activity of other compounds may enhance the antioxidant activity of these extracts, which indicates that phenolic compounds may not be the only or main components resulting in their antioxidant activity^51,57^.

A positive correlation was observed between antioxidant activity and total phenolic content of MPE and UAS/MPE-Ag_2_O QDs*,* indicating phenolic compounds have an important role to play in plant antioxidants^51,58^.

The ability of polar and non-polar extracts to reduce Fe (III) to Fe (II) can be considered as the antioxidant ability of the extract. In the ferric reducing antioxidant potential (FRAP) test, the ferric-tripyridyltriazine (Fe^III^-TPTZ) complex is converted to its ferrous (blue) by antioxidants^59^. According to the FRAP experiment (Fig. 5C), the antioxidant activity results of the samples were found to be in the following order: MPE > UAS/MPE-Ag_2_O QDs as well as FCE > UAS/FCE-Ag_2_O QDs*.* Accordingly, the reducing power of the samples also corresponds to their phenolic compounds concentration. It seems that the reducing power of the extracts may be due to the presence of phenolic compounds^51,58^.

### Antibacterial activity

The antibacterial effect of all studied samples was screened against both gram-negative/positive bacteria using the Disk Diffusion Method (DDM), MIC, and MBC methods. Antibacterial activity was determined by measuring the diameter of the growth inhibition zones of the samples at different concentrations (12.5, 25, and 50 mg/mL) by DDM (Table 1). Each concentration was tested in triplicate. The results showed different levels of antibacterial activity, as well as concentration-dependent antibacterial activity.

**Table 1:** Zone of inhibition (mm) of MPE*,* FCE, Chem-syn/Ag_2_O NPs, UAS/FCE-Ag_2_O QDs and UAS*/*MPE-Ag_2_O QDs samples.

Samples	Concentrations (mg/mL)^a^	*S. aureus*	*E. coli*	*P. aeruginosa*	*E. faecalis*
*M. pulegium* extract (MPE)	12.5	6.5 ± 0.75	8.5 ± 0.24	7 ± 0.59	–
	25	7 ± 0.98	9 ± 0.18	9 ± 0.12	–
	50	7.5 ± 0.15	10 ± 0.13	9.5 ± 0.1	8 ± 0.3
*F. carica* extract (FCE)	12.5	–	7.5 ± 0.4	7 ± 0.12	–
	25	6.5 ± 0.84	8 ± 0.12	8.5 ± 0.27	–
	50	7 ± 0.52	9 ± 0.3	9 ± 0.11	7.5 ± 0.81
UAS/MPE-Ag_2_O QDs	12.5	–	9 ± 0.38	8.5 ± 0.21	–
	25	9.5 ± 0.74	11 ± 0.27	11.5 ± 0.16	–
	50	10 ± 0.53	13 ± 0.10	13 ± 0.32	9.5 ± 0.45
UAS/FCE-Ag_2_O QDs	12.5	9 ± 0.24	10 ± 0.36	9.5 ± 0.41	7 ± 1.27
	25	9.5 ± 0.34	10.5 ± 0.11	12 ± 0.18	9 ± 0.27
	50	12 ± 0.58	12 ± 0.29	13 ± 0.62	10 ± 0.4
Chem-syn/Ag_2_O NPs (uncoated) (control)	12.5	7 ± 1.12	6.5 ± 0.67	9 ± 0.18	–
	25	8 ± 0.31	7 ± 1.19	9 ± 1.3	6.5 ± 1.31
	50	9 ± 0.11	8 ± 0.4	9.5 ± 0.23	7 ± 0.15

^a^Data are expressed as mean ± standard deviation (n = 3).

**Figure 6:**
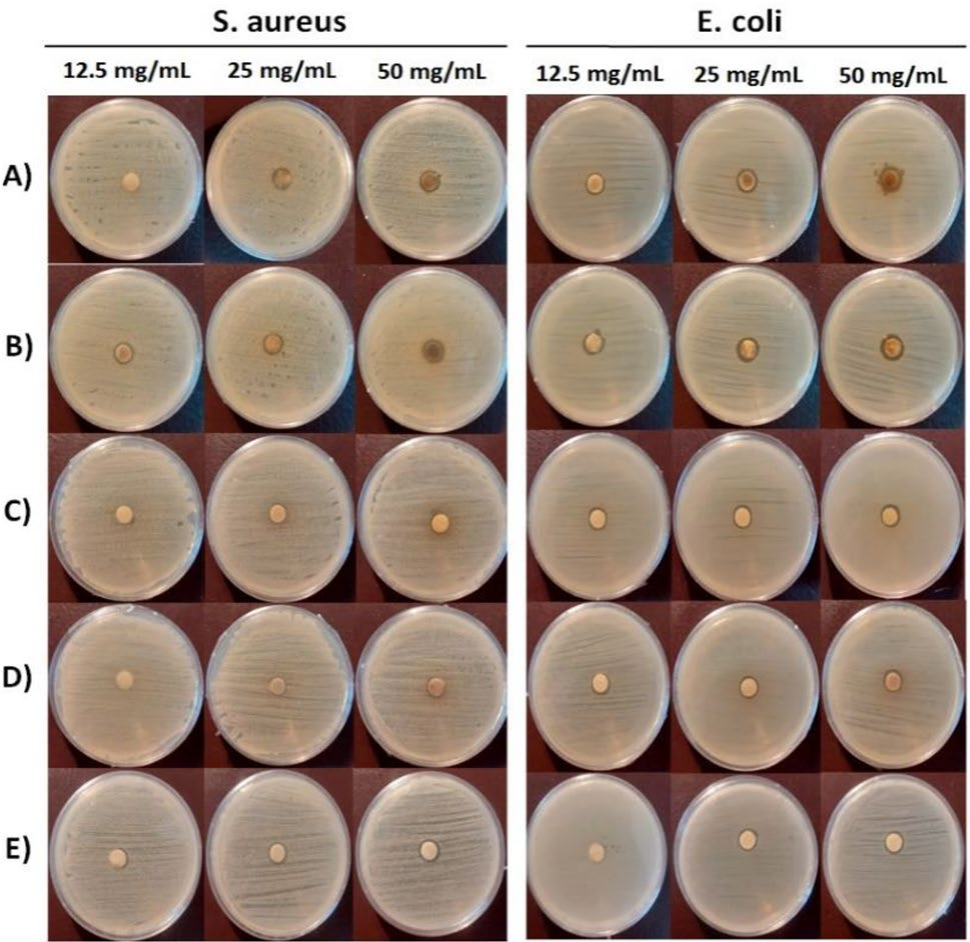
Antibacterial results of UAS/MPE-Ag_2_O QDs (**A**), UAS/FCE-Ag_2_O QDs (**B**), MPE (**C**), FCE (**D**), and Chem-syn/Ag_2_O NPs (Uncoated) (**E**) samples using DDM.

Furthermore, the UAS/phyto-syn/Ag_2_O-NPs had higher antibacterial activity (inhibition zone) than the UA-based extracts for all tested bacteria (Fig. 6A–E). In addition, UAS/MPE-Ag_2_O QDs showed higher antibacterial activity than those prepared by FCE, and MPE has more antibacterial properties than FCE.

UAS/MPE-Ag_2_O QDs have been found to have the highest antibacterial activity among the studied samples. The samples have a more potent effect on gram-negative bacteria than gram-positive bacteria^60^*.* This difference in results is due to the cell wall structure of the bacteria and the permeability of samples to their cell wall^43^.

In order to reveal the antibacterial properties of the samples, MIC and MBC tests were conducted. Based on the MIC results, UAS/MPE-Ag_2_O QDs have the maximum antibacterial activities against *E. coli* and *P. aeruginosa* at the concentrations of 15.6 and 62.5 ppm, respectively (Table 2). Furthermore, the UAS/phyto-syn/Ag_2_O-NPs coated with phytochemicals of plant extracts exhibit better antibacterial properties than plant extracts and the chem-syn/Ag_2_O-NPs.

**Table 2:** Minimal Inhibitory Concentration (MIC) and Minimal Bactericidal Concentration (MBC) (in ppm) of samples against pathogenic bacteria.

Samples	*S. aureus*		*B. subtilis*		*E. coli*		*P. aeruginosa*	
	MIC	MBC	MIC	MBC	MIC	MBC	MIC	MBC
*M. pulegium* extract (MPE)	> 1000	> 1000	125	1000	250	1000	1000	> 1000
*F. carica* extract (FCE)	> 1000	> 1000	250	500	500	500	1000	> 1000
UAS/MPE-Ag_2_O QDs	125	500	125	500	15.6	15.6	62.5	> 1000
UAS/FCE-Ag_2_O QDs	125	500	31.3	250	31.3	250	500	> 1000
Chem-syn/Ag_2_O NPs (uncoated)	500	> 1000	1000	> 1000	500	500	250	250
Cefixime (control)	0.5	2	1	4	2	8	8	32

A comparison of the inhibitory properties (MIC) of quantum dots synthesized by chemical and green methods shows significant differences:

MIC: chem-syn/Ag_2_O-NPs (250–> 1000 ppm) > UAS/phyto-syn/Ag_2_O-QDs (15.6–500 ppm). This increase is due to the presence of soluble phytochemicals responsible for the bio-reduction of silver ions as well as the medicinal properties of the extracts as demonstrated by FTIR and EDX^42^. Likewise, the MBC (minimum bactericidal concentration) values of the plant-synthesized Ag_2_O-QDs showed notable differences with chem-syn/Ag_2_O-NPs. The highest (most effective) MBC test result was related to the UAS/MPE-Ag_2_O QDs at a concentration of 15.6 ppm against *E. coli.* These results indicate a significant advantage in UAS/phyto-syn/Ag_2_O-QDs' antibacterial properties compared to chem-syn/Ag_2_O-NPs.

### Antifungal activity

**Figure 7:**
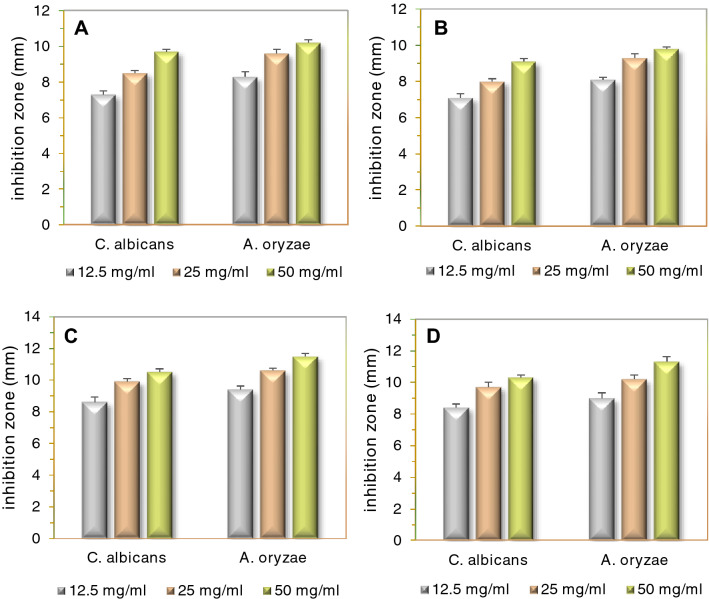
Antifungal activities of MPE*,* FCE (**A**,**B**) and the UAS/MPE-Ag_2_O QDs, UAS/FCE-Ag_2_O QDs samples (**C**,**D**) against two fungi.

In vitro antifungal activities of the UAS/phyto-syn/Ag_2_O-QDs and the aqueous extracts were investigated against *A. oryzae* and *C. albicans* fungal cultures, as the diameter of the inhibition zone (mm) of growth, using the DDT procedure (Fig. 7). Similar to the results of antimicrobial activities, increased concentrations of QDs and extracts enhanced antifungal activity. The UAS/phyto-syn/Ag_2_O-QDs displayed stronger impacts against all the studied fungi than the extracts. Particularly, the UAS/MPE-Ag_2_O QDs with the maximum susceptibility value in *A. oryzae* at 50 mg/mL exhibited the highest antifungal activity against all tested fungal strains.

The phyto-syn Ag_2_O QDs at low concentrations, especially UAS/MPE-Ag_2_O QDs showed higher biological activities than the chem-syn NPs and the tested extracts. It may be due to the extremely small size or high available surface area of the Ag_2_O NPs, which enhances contact and friction and facilitates the penetration of Ag_2_O QDs into the cell through the pores of plasma membrane proteins and causes cell death. Additionally, both the synergistic effect and the extract coating, which increase friction between the microorganisms and phytochemicals in plant extracts, increase the antibacterial activity of plant-synthesized QDs^22,61^.

A comparison was made between the conditions of extraction and synthesis of phyto-Ag_2_O QDs, and those obtained from previously published methods (Table 3). As shown by the results, not only the extraction time was reduced; but also the synthesis of the Ag_2_O QDs with better biological activity (antioxidant, antibacterial, and antifungal) was performed much faster, easier, and with minimal use of toxic chemicals in one step, and under completely green conditions than other Ag_2_O nanoparticles previously reported (Table 3). Ultrasonic irradiation produced pure and extremely fine nanoparticles (quantum dots) with monodispersity in shape and size. Since the properties of nanoparticles depend on their size, it is very imperative to produce monodisperse and pure quantum dots (QDs).

**Table 3:** Comparison of the extraction and nanoparticle synthesis conditions of phyto Ag_2_O QDs with some previously reported methods.

Green nanoparticles	Extraction conditions	Nanoparticle synthesis conditions: using AgNO_3_, corresponding extract and	Diam (nm)	Antibacterial activities (pathogens)		Antifungal activities	Antioxidant activities: DPPH, FRAP, and ABTS assays	Phenol/flavonoid contents	Ref.
				DDM method	MIC and MBC methods				
Ag_2_O NPs/*Pavetta indica* Linn	Pavetta indica Linn leaves, ethanol, 24 h	100–120 °C, 24 h	49.8 nm (width: 169.2 nm)	–	–	–	–	–	^62^
Ag_2_O-guar gum nanocomposite	(1) crude guar gum, boiling ethanol (78 °C), 10 min, washing with (ethanol and acetone) (2) dissolving in distilled water, ultrasonic irradiation, centrifuging and precipitating using cold acetone. 4) dissolving in hot water, centrifuging, precipitating (ethanol), and drying	NaOH, sonication, 30 min	5–20 nm	–	–	–	–	–	^44^
Ag_2_O NPs/Zephyranthes Rosea flower	Petals (Z. rosea flowers), water, 80 °C, 30 min	NaOH, 80 °C, 2 h	10–30 nm	*E. coli, S, mutants, S. aureus*	–	–	DPPH	–	^45^
Ag_2_O NPs/Lippia citriodora leaves	–	Uffle furnace (600 ± 10 °C)	_~ _20 nm	*S. aureus*	*–*	*A. aureus*	*–*	*–*	^6^
Ag_2_O NPs/dragon fruit peel	Dragon fruit peel, water, 100 °C, 15 min	rt, 24 h	25–26 nm	*E. coli, P. aeruginosa, S. aureus*	–	–	FRAP	TFC, TPC	^63^
Ag_2_O NPs/*C. lanceolatus* leaves	Lanceolatus leaves, water, 100 °C, 30 min	Sodium dodecyl sulfate (SDS), 37 °C, dark, 1–3 h	3–25 nm	–	–	–	DPPH	–	^33^
UAS/MPE-Ag_2_O QDs	*M. pulegium* leaves, water, sonication, rt, 10 min	Sonication, rt, 3 min	~ 9 nm	*E. coli, P, aeruginosa, S. aureus, B. subtilis*		*C. albicans, A. oryzae*	DPPH, FRAP, and ABTS tests	TFC, TPC	This work
UAS/FCE-Ag_2_O QDs	*F. carica* fruits*,* water, sonication, rt, 10 min								

**Figure 8:**
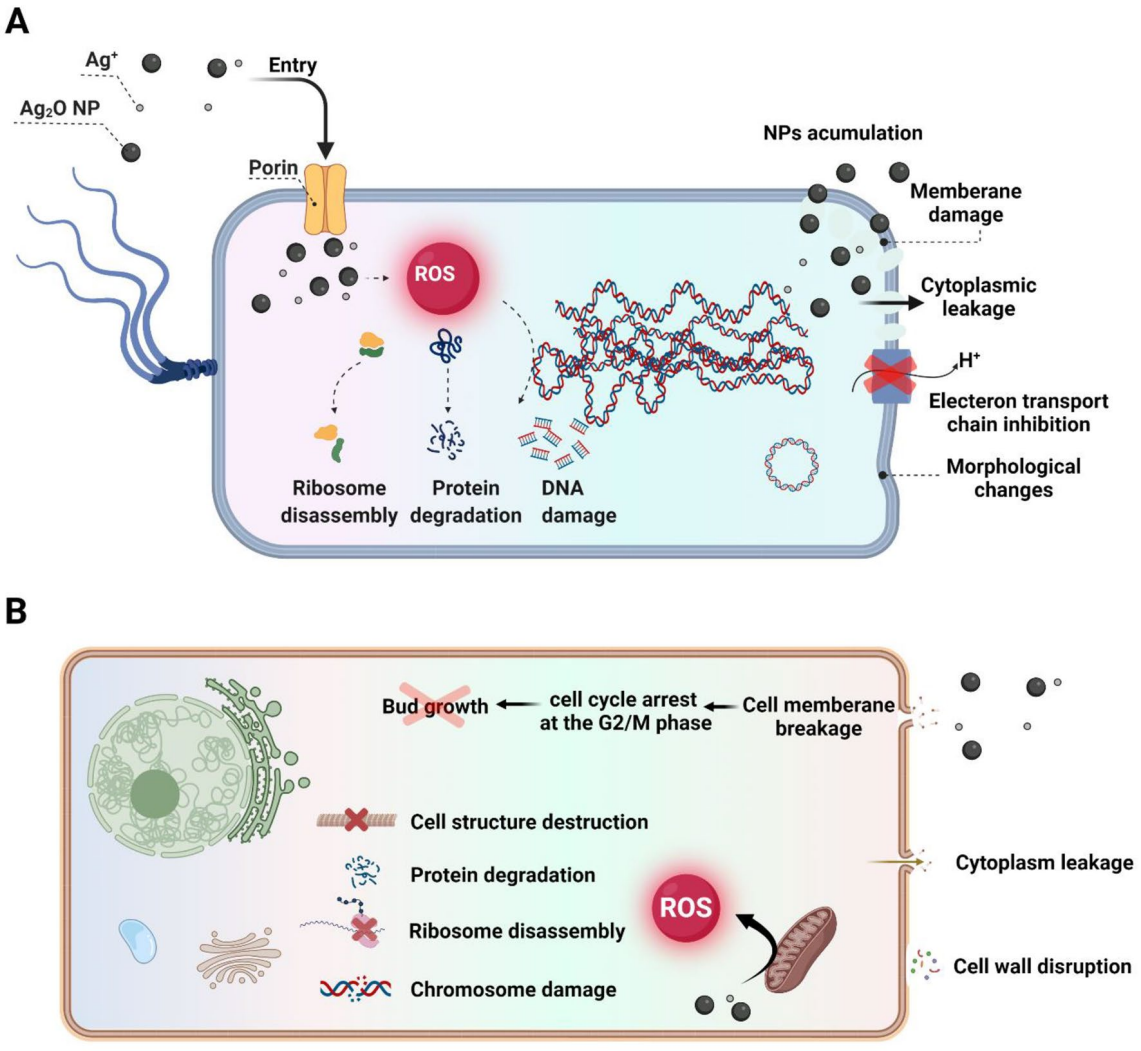
Schematic illustration of antibacterial (**A**) and antifungal (**B**) activity of Ag_2_O nanoparticles.

Ag_2_O nanoparticles' antibacterial properties are due to electrical changes that occur during their interaction with bacterial membranes and increase surface reactivity^64^. There is no clear mechanism for the penetration of Ag_2_O nanoparticles, but bacteria exposed to Ag_2_O NPs showed both morphological changes on the bacterial membrane and disruption of the transport mechanism, which led to significant membrane permeability increases^64,65^. It seems that Ag_2_O NPs, after penetrating into the bacteria, interact strongly with molecules containing phosphorus and sulfur, such as DNA (Fig. 8A). Then damaged DNA loses its ability to replicate, and thus the cell cycle halts at the G2/M phase^64^. Due to the inhibition of ATP synthesis and the production of reactive oxygen species (ROS), cells are exposed to oxidative stress, resulting in the induction of apoptosis. Furthermore, these nanoparticles, after penetrating into bacteria, inactivate bacterial enzymes by releasing ionic (Ag^+^) and atomic (Ag^0^) silver clusters and causing cell death by producing hydrogen peroxide and other free radicals^64^. Silver oxide nanoparticles penetrate the cell wall and cytoplasmic membrane by binding to lipids and proteins, leading to cell lysis and toxic effects inside the cell (Fig. 8)^66^. The mechanisms of antifungal activity of Ag_2_O NPs can be attributed to the production of reactive oxygen species (Fig. 8B). Consequently, these nanoparticles negatively affect the expression of the oxidative enzyme, thereby causing the inability to handle the resulting stress^66^.

Additionally, these nanoparticles can have antifungal properties by disturbing the antioxidant endogenic process and unsettling the pathogen's internal environment. They can also alter homeostatic redox reactions and oxidative stress, leading to an imbalance in osmotic pressure, membrane destruction, and ultimately cell death^66^. These nanoparticles enhance their antifungal effect by inhibiting the enzymatic activity of various enzymes, such as the activity of transferase in lecithin or ATPase in P-glycoprotein ^66^. They arrest the fungal cell cycle by increasing the percentage of cells in G2/M phase and decreasing them significantly in G1 phase^64^.

## Conclusions

This is the first report on fast, eco-friendly, cheap, and completely green synthesis of Ag_2_O QDs using ultrasonic (in both plant extraction and synthesis stages) and plant extracts (*F. carica* fruits and *M. pulegium* leaves). Their antibacterial, antifungal, and antioxidant activities were also examined, and compared with conventional phyto-synthesized Ag_2_O-NPs (with heating and without ultrasound) and chemically synthesized Ag_2_O nanoparticles (plant-uncoated nanoparticles). The use of ultrasonic in synthesis resulted in rapid formation (3 min) of much smaller monodisperse particles (~ 9 nm) than with conventional methods (~ 100 nm). In addition, its use in extraction enhanced both plant cell wall destruction and mass transfer of bioactive compounds into solution. The biological activities results showed that they have good antioxidant, antifungal, and antibacterial properties. As measured by MIC/MBC tests, their antibacterial activities were higher than those of chemically synthesized Ag_2_O-NPs (uncoated), and only small amounts (ppm) of these nanoparticles can inhibit the growth of selected bacteria or kill bacteria (15.62–1000 ppm). Consequently, as the bacteria/fungi studied result in infections and fungal diseases in humans, UAS/phyto-syn/Ag_2_O-QDs are ideal agents for controlling these diseases as well as other pharmaceutical applications. Moreover, their antioxidant properties enable them to neutralize free radicals produced in the body, which contribute to hundreds of dangerous and various diseases. Therefore, they can have industrial and biomedical applications.

## Data Availability

All data generated or analysed during this study are included in this published article.

## References

[1] Abbasi BA (2020). Environmentally friendly green approach for the fabrication of silver oxide nanoparticles: Characterization and diverse biomedical applications. Microsc. Res. Tech..

[2] Rashmi B (2020). Facile green synthesis of silver oxide nanoparticles and their electrochemical, photocatalytic and biological studies. Inorganic Chem. Commun..

[3] Sangappa M, Thiagarajan P (2015). Combating drug resistant pathogenic bacteria isolated from clinical infections, with silver oxide nanoparticles. Indian J. Pharm. Sci..

[4] Torabi S, Mansoorkhani MJK, Majedi A, Motevalli S (2020). Synthesis, medical and photocatalyst applications of nano-Ag_2_O. J. Coord. Chem..

[5] Dharmaraj D (2021). Antibacterial and cytotoxicity activities of biosynthesized silver oxide (Ag_2_O) nanoparticles using *Bacillus **paramycoides*. J. Drug Deliv. Sci. Technol..

[6] Li R (2019). Biosynthesis of silver oxide nanoparticles and their photocatalytic and antimicrobial activity evaluation for wound healing applications in nursing care. J. Photochem. Photobiol. B Biol..

[7] Rajeswari VD (2021). Green synthesis of titanium dioxide nanoparticles using *Laurus nobilis* (bay leaf): Antioxidant and antimicrobial activities. Appl. Nanosci..

[8] Naeimi A, Honarmand M, Sedri A (2019). Ultrasonic assisted fabrication of first MoO_3_/copper complex bio-nanocomposite based on Sesbania sesban plant for green oxidation of alcohols. Ultrason. Sonochem..

[9] Majeed S, Abdullah MSB, Nanda A, Ansari MT (2016). In vitro study of the antibacterial and anticancer activities of silver nanoparticles synthesized from *Penicillium **brevicompactum* (MTCC-1999). J. Taibah Univ. Sci..

[10] Sibiya P, Moloto M (2018). Green synthesis pf Ag_2_S nanoparticles: Effect of PH and capping agent on size and shape of NPs and their antibacterial activity. Dig. J. Nanomater. Biostructures..

[11] Zahedifar M, Shirani M, Akbari A, Seyedi N (2019). Green synthesis of Ag_2_S nanoparticles on cellulose/Fe_3_O_4_ nanocomposite template for catalytic degradation of organic dyes. Cellulose.

[12] Iravani S (2011). Green synthesis of metal nanoparticles using plants. Green Chem..

[13] Hassanpouraghdam MB, Akhgari AB, Aazami MA, Emarat-Pardaz J (2011). New menthone type of *Mentha **pulegium* L. volatile oil from Northwest Iran. Czech J. Food Sci..

[14] Abdelli M, Moghrani H, Aboun A, Maachi R (2016). Algerian *Mentha **pulegium* L. leaves essential oil: Chemical composition, antimicrobial, insecticidal and antioxidant activities. Ind. Crops Prod..

[15] Rad SS, Sani AM, Mohseni S (2019). Biosynthesis, characterization and antimicrobial activities of zinc oxide nanoparticles from leaf extract of *Mentha **pulegium* (L.). Microb. Pathog..

[16] Veberic R, Mikulic-Petkovsek M (2016). Nutritional Composition of Fruit Cultivars.

[17] Soni N, Mehta S, Satpathy G, Gupta RK (2014). Estimation of nutritional, phytochemical, antioxidant and antibacterial activity of dried fig (*Ficus **carica*). J. Pharmacogn. Phytochem..

[18] Mawa S, Husain K, Jantan I (2013). *Ficus **carica* L. (Moraceae): Phytochemistry, traditional uses and biological activities. Evid. Based Complement. Altern. Med..

[19] Saha SK, Chowdhury P, Saini P, Babu SPS (2014). Ultrasound assisted green synthesis of poly (vinyl alcohol) capped silver nanoparticles for the study of its antifilarial efficacy. Appl. Surf. Sci..

[20] Bang JH, Suslick KS (2010). Applications of ultrasound to the synthesis of nanostructured materials. Adv. Mater..

[21] Al Jitan S, Alkhoori SA, Yousef LF (2018). Phenolic acids from plants: Extraction and application to human health. Stud. Nat. Prod. Chem..

[22] Shirazi MS, Foroumadi A, Saberikia I, Farimani MM (2022). Very rapid synthesis of highly efficient and biocompatible Ag_2_Se QD phytocatalysts using ultrasonic irradiation for aqueous/sustainable reduction of toxic nitroarenes to anilines with excellent yield/selectivity at room temperature. Ultrason. Sonochem..

[23] Sullivan KT, Wu C, Piekiel NW, Gaskell K, Zachariah MR (2013). Synthesis and reactivity of nano-Ag_2_O as an oxidizer for energetic systems yielding antimicrobial products. Combust. Flame..

[24] Ercetin T, Senol FS, Orhan IE, Toker G (2012). Comparative assessment of antioxidant and cholinesterase inhibitory properties of the marigold extracts from *Calendula arvensis* L. and *Calendula officinalis* L. Ind. Crops Prod..

[25] Mirzaei A, Mirzaei N, Salehpour Z, Khosravani SA, Amouei M (2013). Phenolic, ascorbic contents and antioxidant activities of 21 Iranian fruits. Life Sci. J..

[26] Re R (1999). Antioxidant activity applying an improved ABTS radical cation decolorization assay. Free Radic. Biol. Med..

[27] Zheleva-Dimitrova D, Nedialkov P, Kitanov G (2010). Radical scavenging and antioxidant activities of methanolic extracts from Hypericum species growing in Bulgaria. Pharmacogn. Mag..

[28] Hudzicki, J. *Kirby-Bauer Disk Diffusion Susceptibility Test Protocol* (2009).

[29] Andrews JM (2001). Determination of minimum inhibitory concentrations. J. Antimicrob. Chemother..

[30] Ravikumar S, Gokulakrishnan R, Raj JA (2012). Nanoparticles as a source for the treatment of fish diseases. Asian Pac. J. Trop. Dis..

[31] Montazerozohori M, Zahedi S, Nasr-Esfahani M, Naghiha A (2014). Some new cadmium complexes: Antibacterial/antifungal activity and thermal behavior. J. Ind. Eng. Chem..

[32] Rokade AA, Patil MP, Yoo SI, Lee WK, Park SS (2016). Pure green chemical approach for synthesis of Ag_2_O nanoparticles. Green Chem. Lett. Rev..

[33] Ravichandran S, Paluri V, Kumar G, Loganathan K, Kokati Venkata BR (2016). A novel approach for the biosynthesis of silver oxide nanoparticles using aqueous leaf extract of *Callistemon **lanceolatus* (Myrtaceae) and their therapeutic potential. J. Exp. Nanosci..

[34] Majeed S, Abdullah MSB, Dash GK, Ansari MT, Nanda A (2016). Biochemical synthesis of silver nanoprticles using filamentous fungi *Penicillium **decumbens* (MTCC-2494) and its efficacy against A-549 lung cancer cell line. Chin. J. Nat. Med..

[35] Bhanjana G (2019). Novel electrochemical sensor for mononitrotoluenes using silver oxide quantum dots. Electrochim. Acta..

[36] Jafarirad S, Mehrabi M, Divband B, Kosari-Nasab M (2016). Biofabrication of zinc oxide nanoparticles using fruit extract of *Rosa canina* and their toxic potential against bacteria: A mechanistic approach. Mater. Sci. Eng. C..

[37] Shume WM, Murthy H, Zereffa EA (2020). A review on synthesis and characterization of Ag_2_O nanoparticles for photocatalytic applications. J. Chem..

[38] Mostafa H, Airouyuwa JO, Maqsood S (2022). A novel strategy for producing nano-particles from date seeds and enhancing their phenolic content and antioxidant properties using ultrasound-assisted extraction: A multivariate based optimization study. Ultrason. Sonochem..

[39] Manikandan V (2017). Green synthesis of silver oxide nanoparticles and its antibacterial activity against dental pathogens. 3 Biotech..

[40] Ibrahim HM (2015). Green synthesis and characterization of silver nanoparticles using banana peel extract and their antimicrobial activity against representative microorganisms. J. Radiat. Res. Appl. Sci..

[41] Majeed S (2022). In vitro evaluation of antibacterial, antioxidant, and antidiabetic activities and glucose uptake through 2-NBDG by Hep-2 liver cancer cells treated with green synthesized silver nanoparticles. Oxid. Med. Cell. Longev..

[42] Steffy K, Shanthi G, Maroky AS, Selvakumar S (2018). Synthesis and characterization of ZnO phytonanocomposite using *Strychnos*
*nux**-vomica* L. (Loganiaceae) and antimicrobial activity against multidrug-resistant bacterial strains from diabetic foot ulcer. J. Adv. Res..

[43] Vinay S, Sumedha H, Nagaraju G, Harishkumar S, Chandrasekhar N (2020). Facile combustion synthesis of Ag_2_O nanoparticles using cantaloupe seeds and their multidisciplinary applications. Appl. Organomet. Chem..

[44] Balachandramohan J, Sivasankar T, Sivakumar M (2020). Facile sonochemical synthesis of Ag_2_O-guar gum nanocomposite as a visible light photocatalyst for the organic transformation reactions. J. Hazard. Mater..

[45] Maheshwaran G (2020). Green synthesis of silver oxide nanoparticles using Zephyranthes Rosea flower extract and evaluation of biological activities. J. Environ. Chem. Eng..

[46] Veberic R, Colaric M, Stampar F (2008). Phenolic acids and flavonoids of fig fruit (*Ficus **carica* L.) in the northern Mediterranean region. Food Chem..

[47] Jebali J (2022). Tunisian native *Mentha **pulegium* L. extracts: Phytochemical composition and biological activities. Molecules.

[48] Spiridon I, Bodirlau R, Teaca C-A (2011). Total phenolic content and antioxidant activity of plants used in traditional Romanian herbal medicine. Cent. Eur. J. Biol..

[49] Phull A-R (2016). Antioxidant, cytotoxic and antimicrobial activities of green synthesized silver nanoparticles from crude extract of *Bergenia **ciliata*. Future J. Pharm. Sci..

[50] Elizabeth M-C (2022). Antioxidant and anti-inflammatory polyphenols in ultrasound-assisted extracts from salvilla (*Buddleja*
*scordioides* Kunth). Ultrason. Sonochem..

[51] Benabdallah A, Rahmoune C, Boumendjel M, Aissi O, Messaoud C (2016). Total phenolic content and antioxidant activity of six wild Mentha species (Lamiaceae) from northeast of Algeria. Asian Pac. J. Trop. Biomed..

[52] Samari F, Parkhari P, Eftekhar E, Mohseni F, Yousefinejad S (2019). Antioxidant, cytotoxic and catalytic degradation efficiency of controllable phyto-synthesised silver nanoparticles with high stability using *Cordia **myxa* extract. J. Exp. Nanosci..

[53] Santos CM, Silva A (2020). The antioxidant activity of prenylflavonoids. Molecules.

[54] Shahidi F, Zhong Y (2015). Measurement of antioxidant activity. J. Funct. Foods..

[55] Racané L (2020). Green synthesis and biological evaluation of 6-substituted-2-(2-hydroxy/methoxy phenyl) benzothiazole derivatives as potential antioxidant, antibacterial and antitumor agents. Bioorg. Chem..

[56] Abdel-Aziz MS, Shaheen MS, El-Nekeety AA, Abdel-Wahhab MA (2014). Antioxidant and antibacterial activity of silver nanoparticles biosynthesized using *Chenopodium **murale* leaf extract. J. Saudi Chem. Soc..

[57] Brahmi F (2017). Antioxidant capacity and phenolic content of two Algerian Mentha species *M. rotundifolia* (L.) Huds, *M. **pulegium* L., extracted with different solvents. J. Complement. Integr. Med..

[58] Waheed I, Ahmad M, Syed N, Ashraf R (2014). Investigation of phytochemical and antioxidant properties of methanol extract and fractions of *Ballota **limbata* (Lamiaceae). Indian J. Pharm. Sci..

[59] Benzie IF, Strain JJ (1996). The ferric reducing ability of plasma (FRAP) as a measure of “antioxidant power”: The FRAP assay. Anal. Biochem..

[60] Haq S (2021). Green synthesis of silver oxide nanostructures and investigation of their synergistic effect with moxifloxacin against selected microorganisms. J. Inorg. Organomet. Polym. Mater..

[61] Nouri A (2020). Ultrasonic-assisted green synthesis of silver nanoparticles using *Mentha aquatica* leaf extract for enhanced antibacterial properties and catalytic activity. Colloids Interface Sci. Commun..

[62] Suresh S, Pradheesh G, Ramani VA (2018). Biosynthesis and characterization of CuO, MgO and Ag_2_O nanoparticles, anti-inflammatory activity and phytochemical screening of the ethanolic extract of the medicinal plant *Pavetta** indica* Linn. J. Pharmacogn. Phytochem..

[63] Phongtongpasuk S, Poadang S, Yongvanich N (2016). Environmental-friendly method for synthesis of silver nanoparticles from dragon fruit peel extract and their antibacterial activities. Energy Procedia..

[64] Allahverdiyev AM, Abamor ES, Bagirova M, Rafailovich M (2011). Antimicrobial effects of TiO_2_ and Ag_2_O nanoparticles against drug-resistant bacteria and leishmania parasites. Future microbial..

[65] Sondi I, Salopek-Sondi B (2004). Silver nanoparticles as antimicrobial agent: A case study on *E. coli* as a model for Gram-negative bacteria. J. Colloid Interface Sci..

[66] Ayanwale AP, Estrada-Capetillo BL, Reyes-López SY (2021). Evaluation of antifungal activity by mixed oxide metallic nanocomposite against *Candida* spp. Process..

